# The pathological significance of selected *MLH1* missense mutations based on the theoretical *in silico* predictions

**DOI:** 10.1186/1897-4287-13-S1-A9

**Published:** 2015-09-09

**Authors:** Mariola Dubanowicz

**Affiliations:** 1 Pomeranian Medical University, Faculty of Medical Biotechnology, Szczecin, Poland

## 

Germline mutations in the human DNA mismatch repair (MMR) genes MSH2 and *MLH1* are associated with the inherited cancer disorder Lynch syndrome (LS). Up to 30% of *MLH1* variants found are missense mutations. The functional consequences in regard to pathogenicity of many of these variants are unclear. Missense mutations affect protein structure or function, but may also cause aberrant splicing. In this study, *in silico* prediction tools (SIFT, PolyPhen-2 and ESEfinder 3.0), literature and an online MMR mutation database review were used to evaluate the clinical significance of 26 MLH1 missense mutations identified in patients from Polish families, suspected of Lynch syndrome. Six *MLH1* VUS were classified as pathogenic, nine *MLH1* missense mutations are classified as neutral. 11 variants require additional research. The results show that *in silico* prediction tools can be utilized in an appropriate and efficient manner to determine the pathogenicity of MMR gene variations.

